# Can the Recording of Motor Potentials Evoked by Transcranial Magnetic Stimulation Be Optimized?

**DOI:** 10.3389/fnhum.2017.00413

**Published:** 2017-08-15

**Authors:** Marco A. C. Garcia, Victor H. Souza, Claudia D. Vargas

**Affiliations:** ^1^Departamento de Biociências e Atividades Físicas, Escola de Educação Física e Desportos, Universidade Federal do Rio de Janeiro Rio de Janeiro, Brazil; ^2^Departamento de Física, Faculdade de Filosofia, Ciências e Letras de Ribeirão Preto, Universidade de São Paulo Ribeirão Preto, Brazil; ^3^Laboratório de Neurobiologia II, Instituto de Biofísica Carlos Chagas Filho, Universidade Federal do Rio de Janeiro Rio de Janeiro, Brazil; ^4^Department of Neuroscience and Biomedical Engineering, Aalto University Espoo, Finland

**Keywords:** transcranial magnetic stimulation, TMS, surface electromyography, sEMG, motor evoked potential, MEP, corticospinal excitability

## Introduction

Transcranial magnetic stimulation (TMS) combined with surface electromyography (sEMG) has been for a long time an important non-invasive tool to investigate and better understand how brain controls the skeletal muscles. However, the present literature still lacks standardization protocols and comprehensive discussions about possible influences of sEMG electrode placement and montages on TMS evoked responses. With the advent of TMS by Barker et al. (1985), several advances have been made in basic and clinical neurophysiology (Rossini et al., 2015). In TMS, a high-intensity brief magnetic pulse applied with a coil over the subject's scalp, induces an electric field across the cortical tissue that depolarizes a group of neuronal pools. Therefore, if a single pulse is applied over a particular spot of the primary motor cortex (M1), the generated action potentials travel down the corticospinal tract reaching a specific muscle or group of muscles, which in turn can be achieved by recording their myoelectric activities. Such myoelectric activity may contain potentials varying from a few micro to millivolts and are recognized as motor evoked potentials (MEPs). MEPs can be recorded by means of sEMG with different electrode types, e.g. surface or indwelling, and montages, e.g. mono and bipolar. Most TMS applications take advantage of MEP amplitude and latency to evaluate the integrity and/or excitability of the motor corticospinal pathway to study normal and abnormal aspects of neurophysiology, including the pathophysiology of many neurological and motor disorders. Some may believe that differences in electrode arrangement for recording MEPs can offer a small impact in data quality; in this case he/she may be a victim of an ordinary pitfall. Thus, we may ask and discuss along this manuscript, what are the disadvantages and advantages of recording MEPs from different surface electrode montages? Do they provide a robust and similar comprehension of motor corticospinal excitability?

## The compound surface EMG signal

Two basic mechanisms regulate muscle force generation from the motor units (MUs): spatial and temporal summation. Considering an increase in muscle force, spatial summation refers to the recruitment of MUs following the size principle (Henneman et al., 1965) while temporal summation accounts to an increase in the firing rate (De Luca and Erim, 1994). Moreover, muscle fibers that belong to a MU may be sparsely—or heterogeneously—clustered throughout the muscle volume (Bodine-Fowler et al., 1990). Consequently, MUs action potentials (MUAPs) recorded over the skin surface may not be uniformly distributed in space as muscle force varies (Merletti and Parker, 2004; Rosa et al., 2008; Garcia and Vieira, 2011; Hodson-Tole et al., 2013). Therefore, sEMG amplitude arising from the algebraic temporal and spatial summation of the action potentials that emanate from the underlying recruited MUs may exhibit different spatial distributions throughout the muscle extent. Previous studies based on mathematical modeling reinforce the need of always considering the inhomogeneous nature of a muscle due to its architecture and MU recruitment when considering the sEMG signal properties (Mesin and Farina, 2005; Messaoudi and Bekka, 2015). Moreover, such properties have been better described in the last years in several superficial muscles with the advent of multi-electrode arrays for high-density sEMG (HD-sEMG) (Holtermann, 2008). For instance, different levels of myoelectric activity may be observed for a same contraction force depending on the site of electrode placement (Rojas-Martinez et al., 2012). In turn, different TMS pulse intensities may lead to distinct distributions of MEP in forearm muscles. Specifically, lower stimulation intensities seem to yield to MEPs spatial distribution like those observed in voluntary contractions (Van Elswijk et al., 2008). Altogether, these few studies exemplify the idea that even though recommendations were conceived to provide robust and representative electrode placement instructions, recording the myoelectric activity under distinct conditions may not be a straightforward task depending on the purpose of the investigation, especially for MEPs elicited with TMS.

## Guidelines for sEMG signal recording: do they fit with MEP acquisition?

Among other methodological issues, there seemed to be lack of consensus regarding the sEMG acquisition and processing since few decades ago (see Zipp, 1982). Consequently, various European groups developed recommendations on how to apply the sEMG. The European concerted action named Surface ElectroMyoGraphy for the Non-Invasive Assessment of Muscles (SENIAM), carried out between the years 1996 and 1999, provides very important guidelines concerning electrode size and placement (see details on http://www.seniam.org). Thereafter, many authors started to follow those recommendations that allowed comparisons among different studies. SENIAM recommendations suggest placing surface electrodes in a bipolar configuration far from the innervation zones (IZ) or tendons as previously detached. They reinforce other methodological issues, such as adopting specific inter-electrode distances to minimize the risk of crosstalk. Hence, one can have some guarantees of recording a sEMG signal with large amplitude and low signal-noise ratio (Hermens et al., 2000). Complementarily, the International Federation of Clinical Neurophysiology (IFCN) published specific recommendations for sEMG recordings in TMS experiments (Rossi et al., 2009; Rossini et al., 2015), which are based on the belly-tendon montage. However, a great variety of approaches have been used in different studies. Some authors have adopted the strategy to place an electrode over the motor point instead of the muscle belly (Carroll et al., 2001; Lefebvre et al., 2004; Garcia et al., 2016; Peres et al., 2017) while others reported to use two electrodes over the muscle belly, i.e., a “conventional bipolar arrangement” (Corneal et al., 2005; Zschorlich and Köhling, 2013). Before starting to proceed to our considerations regarding MEP recording, one might have wondered whether IFCN or SENIAM are suitable for that. Our understanding may be better outlined by the following question: Can corticospinal excitability be suitably evaluated by the protocols of surface electrodes positioning usually adopted by many studies in muscle function and that are in accordance with SENIAM recommendations? We advocate that the answer to this question is PROBABLY NOT, which is partially corroborated by recent findings (Gallina et al., 2017).

## What are the pitfalls and challenges for MEP recording?

Previous general remarks regarding the MUs recruitment under a volitional contraction suggest that the TMS pulse applied over M1 may recruit the same motor units in a similar way (Bawa and Lemon, 1993; Rothwell, 2007). Then, one could infer that recording sEMG signal to assess the motor corticospinal excitability should be conducted by the same protocol as to assess the volitional aspect of a specific muscle group in a motor task. This observation can be a pitfall! Differently to kinesiological and other muscle analysis, we must emphasize that evaluating motor corticospinal excitability with TMS regards the “*global excitability/conductivity of cortical interneurons, fast corticospinal pathways, as well as spinal motoneurons*” (Badawy et al., 2012). Therefore, it may be assumed that would be more suitable to monitor the distal ends of these descending motor pathways at the neuromuscular junctions (NMJ) than from the belly or a different location of a muscle as it is usually suggested (see SENIAM recommendations).

Some particular *key points* related to the mechanisms that underlie MUs recruitment may support the theoretical background of our recommendation. Once the action potentials reach the motor end plates and propagate along the muscle fibers, they will distribute differently throughout the muscle, from both spatial and temporal points of view. Therefore, even though previous studies suggest that muscle fibers might be clustered or distributed in subgroups within the muscle cross-section (Bodine-Fowler et al., 1989, 1990), the action potentials will somehow spread in accordance with the muscle's architecture, function and fiber type. Thus, the sEMG signal will present different amplitudes depending on the overlapping degree of MUAPs, which in turn varies with the relative electrode location (Merletti and Parker, 2004). In contrast, considering that the motor neurons transmit impulses to all corresponding muscle fibers at the motor end plates topographically circumscribed to a particular location, and therefore properly covered by a surface electrode, we may hypothesize that it would lead to a higher probability of a coherent summation of MUAPs trains. Based on this assumption, we may state that recording the sEMG signal from the skin region immediately over the NMJ that is often, but not always, related to the IZ (Barbero et al., 2012), may provide MEPs with lower variance than any other different muscle region. This may be supported by the fact that a surface electrode placed over the IZ/NMJ on a “pseudo-monopolar” montage, would be able to cover the region where a greater population of MUs that innervates a muscle ends and communicates with muscle fibers. Therefore, the electrode can record MEPs derived from the summation of MUAPs that emerge at this region before they propagate along the MUs to distinct muscle locations. This is more evident in skeletal muscles with fibers that do not follow a longitudinal or parallel organization. In this case, MUAPs will propagate with different spatial distribution patterns depending on the MUs territory distributions along the whole muscle (Vieira et al., 2011, 2016). Thus, recording a MEP far from the IZ/NMJ would certainly lead to a lower number of coinciding MUAPs.

Summing up, we support the hypothesis that detecting a MEP from the IZ/NMJ may represent a “maximization” on the MUAPs summation both in time and space of those MUs recruited by a TMS pulse than any other particular region of a muscle. To the best of our knowledge, no previous study has placed this issue in such perspective. Thus, it seems fair to consider that the IZ/NMJ sounds as an interesting and more appropriate location to detect MEPs than any other region over the muscle. Nevertheless, we must pay special attention to those muscles for which the IZ may extend over a larger portion of the muscle length, such as the *vastus medialis* (Saitou et al., 2000; Gallina et al., 2013). It is important to note that choosing the optimal electrode montage also depends on the muscle being studied. Monopolar configurations may be more sensitive to crosstalk. However, crosstalk over the IZ may be less pronounced in intrinsic hand muscles, largely studied in TMS, due to their relatively isolated anatomy. In turn, monopolar montages might represent more precisely the percentage of MUs recruitment in isometric contractions compared to bipolar montages in quadriceps muscle (Rodriguez-Falces and Place, 2016). Additionally, bipolar recordings seem to saturate the M-wave amplitude before the total number of MUs is actually recruited. In this case, MEPs recorded in bipolar configuration during isometric contraction would probably underestimate the amount of neural drive elicited through TMS. Therefore, we recommend that the anatomical properties of target muscle should be taken into account and that the type of electrode configuration to be used should be chosen carefully. Generally, this would mean to overcome, albeit partially, the task of handling the most critical factors that contribute to the resultant sEMG signal, such as muscle architecture and the spatial distribution of MUAPs, as previously discussed. Nonetheless, this would mean that collecting the sEMG signal from a different region that is not related to the IZ/NMJ can induce a misinterpretation of the MEPs, since they may come from a resultant sEMG signal biased by the inhomogeneity of a given muscle.

## Conclusion

Back to the main issues posed in the title of the present manuscript, may different protocols concerning the placement of surface electrodes provide a robust and similar comprehension of motor corticospinal excitability? We would say once more that PROBABLY NOT. It is well known that MEP amplitude, which represents the main parameter considered on evaluating corticospinal excitability, depends on the intensity of TMS pulse shape, electrodes dimension and skull anatomy, among others. Both evoked and volitional MUs recruitments will lead to different profiles of distribution of surface MUAPs, which in turn depends on the territory occupied by those activated MUs. Therefore, in the light of the aforementioned, we conclude that recording a sEMG signal from the corresponding location of IZ/NMJ may supply more reliable MEPs and hence a better comprehension in respect to the motor corticospinal excitability.

## Author contributions

MG Conception, design, and analysis and interpretation of the literature; he has participated in drafting of the manuscript and critical revision of the manuscript for important intellectual content; final approval of the version to be submitted; VS Conception, design, and analysis and interpretation of the literature; he has participated in drafting of the manuscript and critical revision of the manuscript for important intellectual content; final approval of the version to be submitted; CV Conception, design, and analysis and interpretation of the literature; she has participated in drafting of the manuscript and critical revision of the manuscript for important intellectual content; final approval of the version to be submitted.

### Conflict of interest statement

The authors declare that the research was conducted in the absence of any commercial or financial relationships that could be construed as a potential conflict of interest.

## References

[B1] Badawy R. A. B. Loetscher T. Macdonell R. A. L. Brodtmann A. (2012). Cortical excitability and neurology: insights into the pathophysiology. Funct. Neurol. 27, 131–145. 23402674 PMC3812767

[B2] Barbero M. Merletti R. Rainoldi A. (2012). Atlas of Muscle Innervation Zones: Understanding Surface Electromyography and Its Applications. Milan: Springer-Verlag Italia.

[B3] Barker A. T. Jalinous R. Freeston I. L. (1985). Non-invasive magnetic stimulation of human motor cortex. Lancet 11, 1106–1107. 10.1016/S0140-6736(85)92413-4 2860322

[B4] Bawa P. Lemon R. N. (1993). Recruitment of motor units in response to transcranial magnetic stimulation in man. J. Physiol. 471, 445–464. 10.1113/jphysiol.1993.sp019909 8120816 PMC1143970

[B5] Bodine-Fowler S. Garfinkel A. Roy R. R. Edgerton V. R. (1989). Analysis of the spatial distribution of muscle fibers within the territory of a motor unit. J. Biomech. 22, 990 10.1016/0021-9290(89)90130-9 1702521

[B6] Bodine-Fowler S. Garfinkel A. Roy R. R. Edgerton V. R. (1990). Spatial distribution of muscle fibers within the territory of a motor unit. Muscle Nerve 13, 1133–1145. 10.1002/mus.880131208 1702521

[B7] Carroll T. J. Riek S. Carson R. G. (2001). Reliability of the input-output properties of the cortico-spinal pathway obtained from transcranial magnetic and electrical stimulation. J. Neurosci. Methods 112, 193–202. 10.1016/S0165-0270(01)00468-X 11716954

[B8] Corneal S. F. Butler A. J. Wolf S. L. (2005). Intra- and intersubject reliability of abductor pollicis brevis muscle motor map characteristics with transcranial magnetic stimulation. Arch. Phys. Med. Rehabil. 86, 1670–1675. 10.1016/j.apmr.2004.12.039 16084825 PMC3575081

[B9] De Luca C. J. Erim Z. (1994). Common drive of motor units in regulation of muscle force. Trends Neurosci. 17, 299–305. 10.1016/0166-2236(94)90064-7 7524216

[B10] Gallina A. Merletti R. Gazzoni M. (2013). Innervation zone of the vastus medialis muscle: position and effect on surface EMG variables. Physiol. Meas. 34, 1411–1422. 10.1088/0967-3334/34/11/1411 24081116

[B11] Gallina A. Peters S. Neva J. L. Boyd L. A. Garland S. J. (2017). Selectivity of conventional electrodes for recording motor evoked potentials: an investigation with-density surface electromyography. Muscle Nerve 55, 828–834. 10.1002/mus.25412 27649483

[B12] Garcia M. A. C. Catunda J. M. C. Souza M. N. Fontana A. P. Sperandei S. Vargas C. D. (2016). Is the frequency in somatosensory electrical stimulation the key parameter in modulating the corticospinal excitability of healthy volunteers and stroke patients with spasticity? Neural Plast. 2016:3034963. 10.1155/2016/3034963 26881102 PMC4736758

[B13] Garcia M. A. C. Vieira T. M. M. (2011). Surface electromyography: why, when and how to use it. Rev. Andal. Med. Deporte. 4, 17–28.

[B14] Henneman E. Somjen G. Carpenter D. O. (1965). Functional significance of cell size in spinal motorneurons. J. Neurophysiol. 28, 560–580. 14328454 10.1152/jn.1965.28.3.560

[B15] Hermens H. J. Freriks B. Disselhorst-Klug C. Rau G. (2000). Development of recommendations for SEMG sensors placement procedures. J. Electromyogr. Kinesiol. 10, 361–374. 10.1016/S1050-6411(00)00027-4 11018445

[B16] Hodson-Tole E. F. Loram I. D. Vieira T. M. M. (2013). Myoelectric activity along human grastrocnemius medialis: different spatial distributions of postural and electrically elicited surface potentials. J. Electromyogr. Kinesiol. 23, 43–50. 10.1016/j.jelekin.2012.08.003 22967836 PMC3566583

[B17] Holtermann A. (2008). Inhomogeneous Activation of Skeletal Muscles–Investigated by Multi-Channel Surface Electromyography. Dissertation/Doctor's thesis. Trondheim: Norwegian University of Science and Technology.

[B18] Lefebvre R. Pépin A. Louis P. F. Boucher J. P. (2004). Reliability of the motor evoked potentials elicited through magnetic stimulation at three sites. J. Manipulative Physiol. Ther. 27, 97–102. 10.1016/j.jmpt.2003.12.004 14970810

[B19] Merletti R. Parker P. A. (2004). Electromyography: Physiology, Engineering and Noninvasive Applications. Hoboken, NJ: John Wiley & Sons, Inc.

[B20] Mesin L. Farina D. (2005). A model for surface EMG generation in volume conductors with spherical inhomogeneities. IEEE Trans. Biomed. Eng. 52, 1984–1993. 10.1109/TBME.2005.857670 16366222

[B21] Messaoudi N. Bekka R. E. (2015). From single fiber action potential to surface electromyographic signal: a simulation study, in Bioinformatics and Biomedical Engineering 9043, 315-293. Lecture retrieved from Proceedings of International Conference on Bioinformatics and Biomedical Engineering (Cham).

[B22] Peres A. S. C. Souza V. H. Catunda J. M. Y. Mazzeto-Betti K. C. Santos-Pontelli T. E. G. Vargas C. D.. (2017). Can somatosensory electrical stimulation relieve spasticity in post-stroke patients? A TMS pilot study. Biomed. Tech. (Berl.). [Epub ahead of print]. 10.1515/bmt-2016-0162 28475487

[B23] Rodriguez-Falces J. Place N. (2016). Differences in the recruitment curves obtained with monopolar and bipolar electrode configurations in the quadriceps femoris. Muscle Nerve 54, 118–131. 10.1002/mus.25006 26662294

[B24] Rojas-Martinez M. Ma-anas M. A. Alonso J. F. (2012). High-density surface EMG maps from upper-arm and forearm muscles. J. Neuroeng. Rehabil. 9:85. 10.1186/1743-0003-9-85 23216679 PMC3575258

[B25] Rosa I. D. G. Garcia M. A. C. Souza M. N. D. (2008). A novel electromyographic signal Simulator for muscle contraction studies. Comput. Methods Prog. Biomed. 89, 269–274. 10.1016/j.cmpb.2007.10.009 18164097

[B26] Rossi S. Hallet M. Rossini P. M. Pascual-Leone A. Safety of TMS Consensus Group. (2009). Safety, ethical considerations, and application guidelines for the use of transcranial magnetic stimulation in clinical practice and research. Clin. Neurophysiol. 120, 2008–2039. 10.1016/j.clinph.2009.08.016 19833552 PMC3260536

[B27] Rossini P. M. Burke D. Chen R. Cohen L. G. Daskalakis Z. Di Iorio R. (2015). Non-invasive electrical and magnetic stimulation of the brain, spinal cord, roots and peripheral nerves: basic principles and procedures for routine clinical and research application. An update report from an I.F.C.N.Committee. Clin. Neurophysiol. 126, 1071–1107. 10.1016/j.clinph.2015.02.001 25797650 PMC6350257

[B28] Rothwell L. C. (2007). Corticospinal involvement in volitional contractions. J. Physiol. 584, 363. 10.1113/jphysiol.2007.143677 17855750 PMC2277160

[B29] Saitou K. Masuda T. Michikami D. Kojima R. Okada M. (2000). Innervation zones of the upper and lower limb muscles estimated by using multichannel surface EMG. J. Human Ergol. 29, 35–52. 12696320

[B30] Van Elswijk G. Kleine B. U. Overeem S. Eshuis B. Hekkert K. D. Stegeman D. F. (2008). Muscle imaging: mapping responses to transcranial magnetic stimulation with high-density surface electromyography. Cortex 44, 609–616. 10.1016/j.cortex.2007.07.003 18387593

[B31] Vieira T. M. Wakeling J. M. Hodson-Tole E. F. (2016). Is there sufficient evidence to claim muscle units are not localized and functionally grouped within the human gastrocnemius? J. Physiol. 594, 1953–1954. 10.1113/JP271866 27038106 PMC4818604

[B32] Vieira T. M. M. Loram I. D. Muceli S. Merletti R. Farina D. (2011). Postural activation of the human medial gastrocnemius muscle: are the muscle units spatially localized? J. Physiol. 589, 431–443. 10.1113/jphysiol.2010.201806 21115645 PMC3043543

[B33] Zipp P. (1982). Recommendations for the standardization of lead positions in surface electromyography. Eur. J. Appl. Physiol. Occup. Physiol. 50, 41–54. 10.1007/BF00952243

[B34] Zschorlich V. R. Köhling R. (2013). How thoughts give rise to action-conscious motor intention increases the excitability of target-specific motor circuits. PLoS ONE 8:e83845. 10.1371/journal.pone.0083845 24386291 PMC3873337

